# Colon Cancer and Obesity: A Narrative Review

**DOI:** 10.7759/cureus.27589

**Published:** 2022-08-01

**Authors:** Shrimahitha Duraiyarasan, Mayowa Adefuye, Nisha Manjunatha, Vinutna Ganduri, Kruthiga Rajasekaran

**Affiliations:** 1 Research, K.A.P. Viswanatham Government Medical College, Tiruchirappalli, IND; 2 Research, University of Ibadan College of Medicine, Ibadan, NGA; 3 Research, Our Lady of Fatima University College of Medicine, Metro Manila, PHL; 4 Research, Bhaskar Medical College, Hyderabad, IND; 5 Research, Rajah Muthiah Medical College and Hospital, Chidambaram, IND

**Keywords:** estrogen and crc, role of obesity and diabetes, obesity and mutation, ghrelin and crc, resistin and cancer, role of gut microbiomes in cancer, oxidative dna damage, obesity and inflammatory markers, obesity and cancer, colorectal cancer (crc)

## Abstract

Obesity has played a crucial role in the pathogenesis of various cancers, including colorectal cancer (CRC). Obesity has shown to increase the blood levels of insulin, insulin-like growth factor-1 (IGF-1), leptin, resistin, inflammatory cytokines such as interleukin-6 (IL-6), tumor necrosis factor-alpha (TNF-α), monocyte chemoattractant protein-1 (MCP-1) which in turn acts via various signaling pathways to induce colonic cell proliferation and in turn CRC development. It has been shown that estrogen can prevent and cause CRC based on which receptor it acts. Obese patients have relatively low levels of ghrelin and adiponectin that inhibit cell proliferation which further adds to their risk of developing CRC. Obesity can alter the microbial flora of the gut in such a way as to favor carcinogenesis. Weight loss and good physical activity have been related to a reduced incidence of CRC; obese individuals should be screened for CRC and counseled about the importance of weight reduction, diet, and exercise. The best way of screening is using BMI and waist circumference (WC) to calculate the CRC risk in obese people. This study has reviewed the association between obesity and its pathophysiological association with CRC development.

## Introduction and background

Colon cancer has been associated with increased cancer-related mortality and is most prevalent in the western parts of the world [1]. The maximum number of cases have been seen in North America, Europe, New Zealand, and Australia, while the lowest was in Africa [1]. The number of diseased individuals has been on the rise in the United States of America, and an increased incidence has been associated with a lowered socioeconomic status [2,3]. Environmental, genetic, and nutritional factors play a vital role in the pathogenesis of colorectal cancer (CRC) [3]. Few genes and pathways associated with colon cancer are nuclear factor kappa B (NF-κB), mitogen-activated protein kinase (MAPK) pathways, leptin, cyclin D, matrix metalloproteinases (MMPs), signal transducer and activator of transcription-3 (STAT-3) genes, chromosomal instability pathway (CIN), and DNA mismatch repair system (MMR) [4]. Nutritional factors, such as vitamin D, dietary fiber, whole grains, etc., have declined the incidence of CRC [3]. At the same time, alcohol, obesity, insulin resistance, and processed meat are associated with a higher risk of colon cancer [3]. Obesity can increase an individual’s risk of developing various tumors related to chronic inflammation and hypoxia, resulting in neovascularization, which predisposes them to cancer [5]. The majority of CRC (41%) occurs at the proximal part of the colon, the second most common location of CRC is the rectum (28%), and the third is the distal colon (22%) [3]. Many societies have framed guidelines for screening of CRC; two such important guidelines are (1) the joint guidelines from the American Cancer Society, the United States multi-Society Task Force on Colorectal Cancer [6] and (2) the United States Preventive Services Task Force guidelines [7]. Colonoscopy is the gold standard for diagnosing CRC, whereas staging of CRC is done using computerized tomography (CT) [3]. Tumor size (T), regional lymph node (N), and metastasis (M) is the TNM method of staging colon cancer [3]. Carcinoembryonic antigen (CEA) can be used to monitor treatment response and recurrence of tumors [3]. Treatment options may vary based on the disease severity of CRC; localized CRC is treated with surgical resection, lymph node metastasis can be treated with adjuvant chemotherapy, while chemotherapy is the best treatment for patients with advanced disease [8]. Many studies have shown that the westernization of diet and energy-rich food can lead to the onset of obesity in childhood which is shown to be highly associated with CRC. Since it is hard to achieve diet control in any age group, preventing CRC in obese patients can be challenging. The focus of this review article was to divulge the association between CRC and obesity, thus explaining the pathophysiology, preventive methods, screening methods, diagnostic modalities, and treatment options for CRC.

## Review

Obesity has been linked to CRC in several studies. Obesity can be assessed using the body mass index (BMI), and the relative risk of obese patients getting CRC is higher in males when compared to females [9,10]. About 30-70% of CRC has been linked to obesity (BMI ≥ 30 kg/m^2^) and overweight (BMI ≥25-29.9 kg/m^2^) [9,10]. Abdominal obesity is seen to have a higher association with CRC than subcutaneous fat deposition [9,10]. Several studies show that the early onset of obesity has been associated with a higher incidence of CRC [11]. Though the mechanisms that connect obesity and CRC have not been fully understood, the most critical pathophysiological mechanisms are explained here. Keimling et al. conducted a study among 203,177 people, including males and females, for ten years to determine the association between the body mass index (BMI), waist circumference (WC), and hip circumference [12]. After a follow-up of 10 years, 2869 of them had developed CRC [12]. The study inferred that the increased BMI and WC are associated with an increased risk of CRC, but this association was seen in male patients only [12]. A population-based cohort study conducted by Li et al. among 134,225 people for 11 years in females and 5.5 years in males from China concluded that 935 people had developed CRC [13]. The study postulated that general and central obesity was associated with a risk of CRC, but the risk was only significant in males [13]. Wang et al. conducted a study involving 95151 people, of which 953 developed CRC at the end of eight years which deduced that increased BMI and WC are associated with an increased risk of CRC in both males and females [14]. A similar study was conducted by Maclnnis et al., which involved 1556 males and revealed the prevalence of colon cancer in 153 patients (Table 1) [15].

**Table 1:** Population table explaining the association between obesity and CRC CRC: colorectal cancer; BMI: body mass index; WC: waist circumference

Reference	Duration	Study location	Study design	Number of people in the control group	Number of CRC cases	Conclusion
Keimling et al. [12]	10 years	-	-	203,177	2869	Increased BMI and WC were associated with colon cancer in males.
Li et al. [13]	11 years-female 5.5 years -male	China	Cohort	124,225	935	Central obesity and general obesity correlated to colon cancer in males.
Wang et al. [14]	8 years	-	Prospective cohort study	95,151	953	In both male and female populations, increased BMI and WC were associated with colon cancer.
Maclnnis et al. [15]	-	-	-	16,556	153	Obesity led to colon cancer in male patients.

Type two diabetes mellitus and CRC

Obesity has been linked to type 2 diabetes, which has been linked to CRC [16]. Higher insulin levels and insulin-like growth factor (IGF-1) are associated with an increased proliferation of colon cells, resulting in malignancy [16]. The risk is even higher in patients using diabetic medications like sulfonylureas and insulin [16]. Increased glycated hemoglobin levels have also strongly predicted the poorest clinical outcome in CRC patients [17]. Milek et al. conducted a study involving 976 people who had their colonoscopies done previously; the results showed that people with diabetes mellitus developed colonic polyps and cancers more often than people without diabetes mellitus [18]. Increased insulin and glucose can cause translocation and upregulation of Rho-associated protein kinase 1 (ROCK-1), which activates Pproliferating cell nuclear antigen (PCNA) and causes centrosome amplification, which tends to favor carcinogenesis [19,20]. 

Insulin and insulin-like growth factors in CRC development

Insulin, insulin-like growth factor (IGF), insulin receptor (IR), signaling pathways, and IGF-binding protein led to cell proliferation and inactivation of apoptosis, enhancing the process of carcinogenesis [21]. The levels of insulin and IGF are influenced by various factors such as diabetes mellitus, acromegaly, excess energy, hypertriglyceridemia, dietary pattern, obesity, etc. [22]. In the course of CRC, many pathways are implicated. Insulin and IGF are overexpressed in obese patients, leading to activation of the PI3K/Akt signaling pathway that leads to increased survival and growth of cells, amplifying carcinogenesis [23]. Src is a protein oncogene (protein tyrosine kinase) that increases cell growth, proliferation, survival, and migration [24-26]. It has many domains (SH2, SH3, regulatory tails, etc.) in an inactivated form in normal cells, and tends to induce phosphorylation and activation of the PI3K/Akt pathway when activated, promoting the development of CRC [24-26]. IGF-1 causes cytoplasmic degradation of P53 (a tumor suppressor gene), which leads to uncontrolled cell proliferation and neoplasia [27].

Leptin and CRC development

Leptin contributes to the pathophysiology of obesity-related CRC by disrupting signaling pathways at the colon’s adipokine receptor. Leptin is a part of a wide range of protein substances called adipokines, predominantly produced by the adipose tissue [28,29]. It activates different pathways (signal transducer and activator transcription, mitogen-activated protein kinase, PI3K), favors angiogenesis, increases cell proliferation and growth, and inhibits apoptosis, thereby playing a crucial role in the carcinogenesis of CRC [28,29]. Leptin can induce several tissue responses, as shown by studies that indicate that the expression of the leptin receptor varies between the different subsets of CRC [30]. The soluble leptin receptor (sOB-R) might be implicated in leptin’s functional control. According to a case-control study conducted by Aleksandrova et al. in Europe that studied 1129 patients with CRC and 1129 controls, it was deduced that the level of sOR-R in the blood is inversely related to the occurrence of colon cancer [31]. Since leptin is released from adipose tissues, it can be overexpressed in obese patients [32,33]. It mediates satiety by acting via the Ob receptor, which in turn activates a cascade of signaling pathways. Obese people who overexpress leptin may develop resistance to the hormone [32,33]. Obesity also activates the suppressor of cytokine signaling-3 (SOCS-3), which lowers the sensitivity of the vagal nerve’s afferent branch, encouraging carcinogenesis [32,33].

Adiponectin and CRC

Adiponectin is a protein hormone released from adipocytes, and it is inversely proportional to the adipocyte level; thus, its levels are lowered in obese people [34]. It can stimulate the adenosine monophosphate-activated protein kinase (AMPK) pathway, which inhibits cell proliferation and slows the progression of CRC [34]. Homeostasis of cell growth is maintained mainly by adiponectin [35]. When the colonic epithelium is exposed to any carcinogen in the presence of reduced circulating adiponectin, there is an elevated risk of developing CRC [35]. It functions via adiponectin receptors 1 and 2 in both CRC and normal colonic mucosa [36]. It also plays a role in glucose regulation by sensitizing insulin, activating the cell death cascade by increasing the activation of P53 and Bax and inhibiting Bcl2, thus conferring protection against CRC [37].

Role of ghrelin in CRC development

Most of the ghrelin is produced by the stomach, but a small amount is secreted by the small intestine [38,39]. It is not only a characteristic of normal cells, but even cancer cells are capable of producing ghrelin. It stimulates the growth hormone release via the growth hormone secretagogue receptor (GHS-R) [38,39]. It helps improve weight loss due to cancer or other significant diseases by maintaining the energy balance [38,39]. Furthermore, it activates various pathways (RAS, PIK-3 kinases, mammalian target of rapamycin, Akt, etc.), which play a crucial role in CRC development [38,39].

Resistin and CRC development

**Figure 1:**
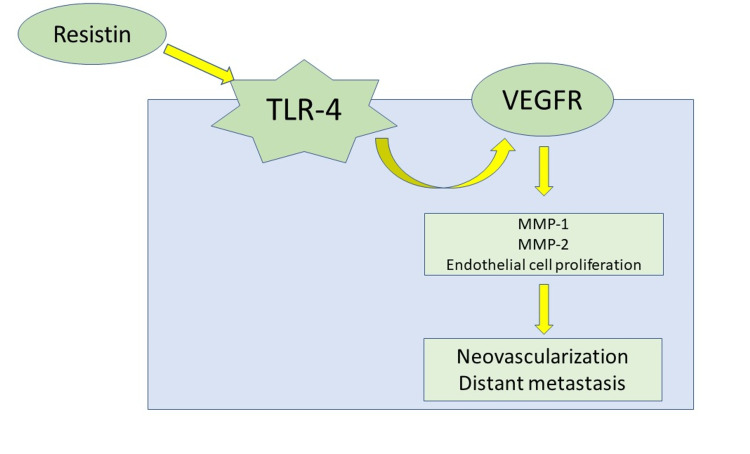
Role of resistin in carcinogenesis The image is created by the author (Shrimahitha Duraiyarasan) of this study. TLR-4: Toll-like receptor-4; VEGFR: vascular endothelial growth factor receptors; MMP-1: matrix metalloproteinases-1; MMP-2: matrix metalloproteinases-2

Resistin, another hormone released from the adipocytes, is elevated in patients with CRC [40]. Toll-like receptor-4 (TLR-4) plays a vital role in identifying various bacterial and viral structures, activates multiple immune responses, and releases cytokines, thereby helping the host fight against the microbes [41,42]. Resistin can compete with the lipopolysaccharide molecules to bind and activate the TLR-4, thereby increasing the inflammatory response [41,42]. Resistin also increases the number of vascular endothelial growth factor receptors (VEGFRs), matrix metalloproteinases 1 and 2 (MMP-1 and MMP-2), which stimulates proliferation of endothelial cells and hence helps angiogenesis (Figure 1). Therefore, resistin plays an essential role in the pathogenesis of CRC by activating the inflammatory pathway and promoting angiogenesis [43].

Variable effect of estrogen on CRC development

**Figure 2:**
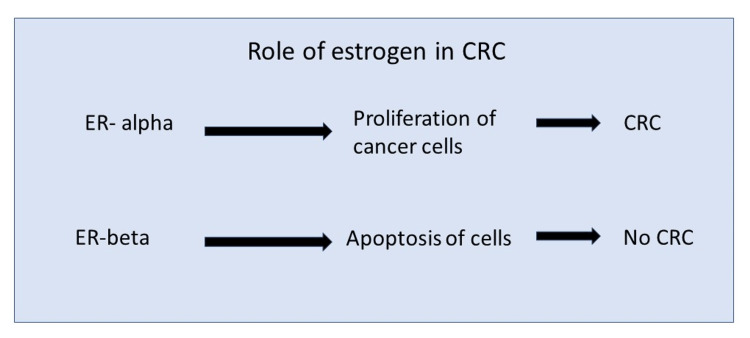
Estrogen receptors and CRC development The image is created by the author (Shrimahitha Duraiyarasan) of this study. ER-alpha: estrogen receptor-alpha; ER-beta: estrogen receptor-beta; CRC: colorectal cancer

Obese patients show increased estrogen levels due to the peripheral conversion of androgens to estrogen in adipocytes. This process is mediated by two receptors, namely estrogen receptor alpha (ER-alpha) and estrogen receptor beta (ER-beta). ER-beta causes cell death in CRC cells, whereas ER-alpha favors the rapid multiplication of CRC cells (Figure 2). ER-beta is the predominant receptor in the colon, and increased estrogen level in obesity confers protection against CRC via the receptor itself [44]. ER-beta activation also increases the DNA repair mechanisms and has anti-inflammatory effects by reducing the level of interleukin-6 (IL-6) [44]. In patients with CRC, the ER-alpha predominates even in obese people, making estrogen a positive factor in favoring the development of CRC in later stages of life [44]. Postmenopausal hormone replacement therapy (HRT) has reduced CRC incidence, explaining the protective effect of estrogen in CRC [45,46]. 

Role of inflammation in CRC

Obese patients have plenty of macronutrients stored in their fat cells that stimulate the release of inflammatory cytokines, such as interleukin-6 (IL-6), tumor necrosis factor-alpha (TNF-α), and matrix metalloproteinase-9 (MMP-9); IL-6 can increase circulating C-reactive protein in the blood [47,48].

IL-6 is mainly produced by the fibroblast cells, and it stimulates the fibroblasts to make vascular endothelial growth factor (VEGF) which mediates neovascularization and thus favors carcinogenesis in CRC [49]. Studies show that IL-6 and C-reactive protein can be used as diagnostic tools to determine the further progression and evaluate the CRC’s prognosis [50]. It is seen that transforming growth factor-beta (TGF-β) acts via IL-6 to activate the signal transducer and activator of transcription-3 (STAT-3) in the tumor cells, which requires the soluble IL-6 receptor present in the tumor cells [51]. IL-6 activates glycoprotein-130 (gp-130) and also has a positive effect on a few signaling pathways like STAT-3 and Janus kinases (JAKs) [52]. TNF-α plays a role in CRC pathogenesis via the Wnt/β-catenin pathway and PI3K/AKT pathway [52]. The Wnt signaling pathway includes various proteins like cyclin-D1, c-Myc, and epithelial-mesenchymal transition-related proteins, which are involved in the formation of cancers [53,54]. TNF-α can exhibit varying effects on the tumor microenvironment (TME) and hence be responsible for tumor progression and suppression [55]. The tumor microenvironment (TME) is the support system for the cancer cells that favors cancer cells in metastasis and growth [56]. TME includes various immune cells infiltrating cancer like lymphocytes, fibroblasts, neovascularization, lymphatics, fat cells, and extracellular matrix [56]. It can exhibit antitumor effects by acting at the TNF receptor 1 and 2 (TNFR1 and TNFR2), which work through various signaling pathways [57]. The permeability of the blood vessels increases, resulting in the migration of the immune cells into the tumor location and destruction of the tumor’s blood vessels, making it an effective mode of treatment in cancer patients [57]. 

The adipocytes secrete the pro-inflammatory cytokine monocyte chemoattractant protein-1 (MCP-1) [58,59]. Macrophages are the predominant immune cells that can enter cancer cells [60,61]. The cancer cells are the primary cells that release MCP-1, which plays a vital role in the inflammatory response at CRC by bringing the monocytes into the tumor location, enhancing the process of carcinogenesis [60,61].

Gut microorganisms and CRC

Various microorganisms, including bacteria, viruses, and fungi, collectively called the human microbiota, exist typically inside the human body [62]. Any alteration in their normal homeostasis can lead to various diseases such as obesity and can progress to the formation of the CRC [62]. Bacteroides fragilis toxin has been associated with CRC development [63]. Normal healthy colonic mucosa is not rich in fusobacterium nucleatum [64]. Yet, it has been isolated in many CRC patients, and studies have shown that it localizes to the colon through the bloodstream [64]. The miRNA is either produced at the gut epithelium or absorbed from the food consumed [65,66]. Adequate miRNA levels control the transcription of various bacteria such as fusobacterium, inhibiting their growth [67]. A reduced number of miRNAs alter the microbiota leading to CRC development [67]. The lipopolysaccharide (LPS) has been seen to activate the nuclear factor kappa B (NF-kB) pathway and increase their potential to invade distant tissues [68].

Obesity and mutation

Fat metabolism happens at the cellular level through various processes, one of which is lipid peroxidation [69]. Lipid peroxidation produces many free radicals like malondialdehyde (MDA), which damages cells, acts as a genotoxic substance, enhances precancerous conditions by cross-linking DNA, and increases the potential to develop CRC [70,71]. 4-Hydroxy-2-nonenal (4-HNE) is another mutagenic substance produced by lipid peroxidation and plays an essential role in oxidative stress [72].

Obesity and angiogenesis

Obesity raises blood levels of vascular endothelial growth factor (VEGF), angiopoietin, angiogenin, and the VEGF receptor, all implicated in angiogenesis [73]. Adipocyte formation is accompanied by new blood vessel formation, mediated by MMP, fibrinolytic and proteolytic substances, followed by endothelial cell proliferation leading to new blood vessel formation [74].

Oxidative stress

Obesity promotes the development of CRC and the formation of reactive oxygen species (ROS) [75,76]. ROS is necessary to maintain good cell function, but if present in abundance, it can lead to detrimental effects since it favors CRC; ROS can cause DNA breaks at locations such as the tumor suppressor gene or oncogenes, which destroys the proteins responsible for regulating cell growth and proliferation, thus leading to the development of various cancers [75,76]. Obesity can cause chronic inflammation and increase the level of leptin, activation of protein kinases, activation of polyol pathways, and other mechanisms that can elevate the oxidative stress inside the cell [77].

Prevention

**Figure 3:**
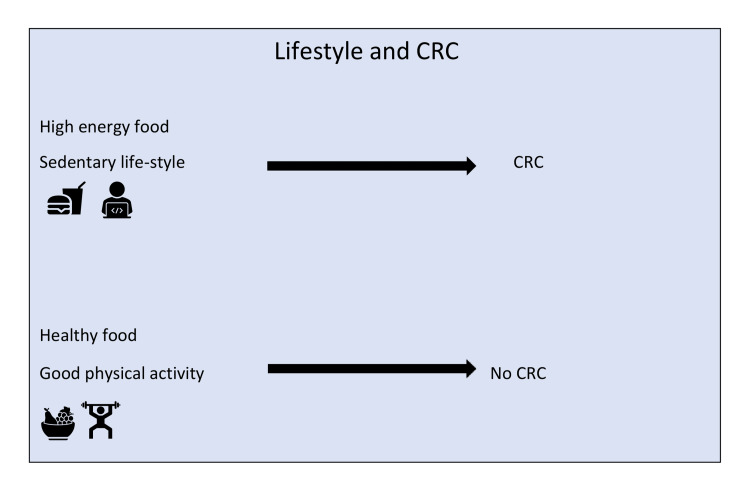
Role of lifestyle in CRC development The image is created by the author (Shrimahitha Duraiyarasan) of this study. CRC: colorectal cancer

In obese people, diet is critical in avoiding the development of CRC. Cabbage, broccoli, onions, ginger, wheat, honey, turmeric, a few herbs, carrots, and berries are among the foods that can help prevent CRC [4]. Most food substances raise the antioxidant level and help reduce obesity-associated CRC [4]. Studies show that if people exercise and improve other forms of physical activity, the probability of developing CRC and other cancers reduces significantly (Figure 3) [78]. Obese people who lose weight have a lower risk of developing CRC [79]. In CRC patients, increased physical activity has also been shown to reduce mortality and side effects of cancer treatment and improve postchemotherapy recovery [80]. Samad et al. conducted a meta-analysis based on 19 cohorts and a few case-control studies, which concluded that a statistically significant reduction in the risk of developing CRC is seen in physically active males and females [81].

Limitations

This study explains various mechanisms that cause CRC in obese people, few of which will require further studies to be proven. Diet and physical activity modification play a vital role in the development of CRC; hence can be used as a target for treatment and prevention. But the part of medical management is not discussed in this study.

## Conclusions

One of the main determinants for developing CRC is having a BMI corresponding to overweight or obesity. This review article elaborates on the association between obesity and CRC and explains the risk factors and common mechanisms involved in CRC pathogenesis. Its pathogenesis is mainly mediated by obesity-associated hyperinsulinemia, increased circulating levels of leptin, resistin, and various cytokines by altering the gut microbial flora and oxidative stress. Thus, physicians should regularly screen obese individuals to pick up early cases of changes in colonic mucosa to prevent CRC development in the future. We should formulate new guidelines based on the BMI, WC, body fat distribution (central or general obesity), and total body fat to aid in the early and easy screening of CRC risk in obese people. Patients should be encouraged to reduce weight through a healthy diet and regular physical activity since weight loss can prevent CRC development. It can also reduce mortality in obese people who have already developed CRC. Thus, weight loss is the primary modality for avoiding and reducing mortality of obesity-associated CRC. In the future, we need more in-depth research about this topic to understand the association between obesity and CRC, screening, and treatment. 
